# Forced Normalization Revisited: New Concepts About a Paradoxical Phenomenon

**DOI:** 10.3389/fnint.2021.736248

**Published:** 2021-08-27

**Authors:** José Augusto Bragatti

**Affiliations:** Clinical Neurophysiology Unit, Service of Neurology, Hospital de Clínicas de Porto Alegre, Porto Alegre, Brazil

**Keywords:** epilepsy, psychiatric comorbidities, forced normalization, kindling, EEG/fMRI

## Abstract

The phenomenon of Forced Normalization (FN) was first described by Landolt in 1953, who described the disappearance of epileptiform discharges in the EEG of patients with epilepsy, concomitant with the development of psychotic symptoms. Later, Tellenbach coined the term “alternative psychosis” referring specifically to the alternation between clinical phenomena. Finally, in 1991, Wolf observed a degenerative process involved in the phenomenon, which he called “paradoxical normalization.” Initially, FN was explained through experimental models in animals and the demonstration of the kindling phenomenon, in its electrical and pharmacological subdivisions. At this stage of research on the epileptic phenomenon, repetitive electrical stimuli applied to susceptible regions of the brain (hippocampus and amygdala) were considered to explain the pathophysiological basis of temporal lobe epileptogenesis. Likewise, through pharmacological manipulation, especially of dopaminergic circuits, psychiatric comorbidities began to find their basic mechanisms. With the development of new imaging techniques (EEG/fMRI), studies in the area started to focus on the functional connectivity (FC) of different brain regions with specific neuronal networks, which govern emotions. Thus, a series of evidence was produced relating the occurrence of epileptic discharges in the limbic system and their consequent coactivation and deactivation of these resting-state networks. However, there are still many controversies regarding the basic mechanisms of network alterations related to emotional control, which will need to be studied with a more homogeneous methodology, in order to try to explain this interesting neuropsychiatric phenomenon with greater accuracy.

## Introduction

The bidirectionality between epilepsy and mental disorders began to be observed in the middle of the nineteenth century, and this concept has evolved since then (Kanner et al., 2017). Initially described from the observation of patients with epilepsy and psychotic symptoms, this association later expanded to other neuropsychiatric disorders, such as depression, conversion disorder and migraine (Krishnamoorthy and Trimble, 1999; Russo et al., 2017). At the beginning of the twentieth century, several studies on the biological antagonism between epilepsy and schizophrenia flourished, which culminated in the development of convulsive therapy, introduced by von Meduna, for the treatment of psychotic patients (Schmitz, 1998).

Inspired by these works, Landolt, in 1953, introduced the concept of FN, referring to the disappearance of epileptiform activity in the EEG of patients with drug-resistant epilepsy concomitant with an acute behavioral change. In the following decade, Tellenbach, applying the concepts of his predecessor to purely clinical aspects, called “alternative psychosis” the mental manifestations that alternate with the seizures of these patients. Finally, in 1991, observing that one of the facets of this phenomenon, in spite of the EEG normalization, was a deterioration of the clinical picture, Wolf proposed the term “paradoxical normalization” (Calle-López et al., 2019).

The diagnostic criteria for FN include an established diagnosis of epilepsy and the presence of an acute/subacute behavioral disorder, with a concomitant reduction in epileptic activity on the EEG, and/or complete cessation of clinical seizures for at least 1 week (Krishnamoorthy and Trimble, 1999). In a recent systematic review, Calle-López and colleagues described the most typical aspects of FN: it occurs predominantly in patients with long-term, uncontrolled epilepsy; the most common psychiatric manifestation is psychosis; and triggering factors are antiseizure medications (ASM) and epilepsy surgery. Discontinuation of the implied ASM results in complete resolution of the FN, although use of antipsychotics does not guarantee a good evolution (Calle-López et al., 2019).

The aim of this mini-review is to report the evolution of the various pathophysiological mechanisms evidenced by the development of research methods over the years, and to discuss controversies and gaps on the subject, proposing future studies on this old-new neuropsychiatric phenomenon.

## Biological Antagonism: Electrical and Pharmacological Kindling Phenomena

Slater et al. (1963) deepened Landolt’s concepts by describing a special form of psychosis associated with epilepsy (schizophrenia-like), separating it from the classic form of schizophrenia, due to its characteristic preservation of the individual’s affection and capacity for interpersonal relationships. In this sense, the Psychosis of Epilepsy closely resembles the amphetamine psychosis described by Bell (1965). Further, corroborating Landolt’s concepts, Flor-Henry (1969) established a series of correlations between psychosis and epilepsy. Amongst them, (1) an association of psychosis and a temporal epileptic focus involving the dominant hemisphere; (2) associations between left temporal lobe epilepsy with schizophrenia, right temporal lobe with manic-depressive illness, and bilateral foci with schizoaffective states; and finally, (3) inverse correlations between frequency of temporal lobe seizures and psychotic symptoms, and frequency of generalized seizures with manic-depressive syndrome.

One of the pioneering methods in investigating the pathophysiology of epilepsies, the animal models of epileptic seizures have revealed seminal knowledge about the mechanisms underlying comorbidities and their bidirectionality, as well as their shared genetic predisposition. Animal models have contributed greatly to the current recognition of epilepsy as a spectrum disorder, and also to the identification of potential targets for its treatment and prevention (Brooks-Kayal et al., 2013).

One of the most used experimental models is the kindling phenomenon. There are two forms of kindling: (1) electrical kindling; and (2) pharmacological kindling. In the electrical kindling method, through a controlled process of electrical stimulations, a predictable sequence of gradual changes in the molecular and cellular organizations of neuronal circuits is produced. Thanks to this excellent experimental model for temporal lobe epilepsy, we now understand the consequences of repeated epileptic seizures, and can see more clearly that their effects are not benign (Sutula and Kotloski, 2017). The pharmacological (or dopaminergic) kindling studies the chronic effects of amphetamines, cocaine, lidocaine and dopaminergic agonists, and is a great experimental model for behavioral changes such as psychosis (Pollock, 1987).

Goddard et al. (1969) defined the pathophysiological bases of electrical kindling. The authors found that by applying low-amplitude and high-frequency electrical stimuli to susceptible brain regions (amygdala, hippocampus, septum), a permanent state of neuronal excitability is established, with gradual progression of behavioral and EEG responses, culminating in a full motor seizure. They proposed that the mechanism could be a progressive lowering of the afterdischarge threshold, caused by the serial electrical stimuli, with accumulation of afterdischarges, until the development of a complete seizure. There is also a transfer of excitation to the contralateral amygdala (mirror focus), and once this new focus has been established, the ability of the original focus to produce a seizure after electrical stimuli becomes diminished (McIntyre and Goddard, 1973). Thus, the secondary focus inhibits the development of seizures originating in the primary focus.

Pharmacological kindling develops through the application of pharmacological agents (amphetamines, cocaine, lidocaine) in the limbic system of rodents, with the production of chronic mental effects (stereotypes, disturbed learned behavior, paranoid psychosis). It differs from electrical kindling as its end point is some kind of affective expression, as well as by the enduring persistence of its effects (Lesse and Collins, 1979). The phenomenon is modulated by changes in dopaminergic pathways and dopamine receptors, vasopressin and endogenous opiate peptides (Post et al., 1984), which are modified by stress (Ellinwood and Kilbey, 1980).

An interesting study developed a model of psychosis using associated electrical and pharmacological stimuli (Stevens and Livermore, 1978). Through this combined method, the authors inhibited the release of dopamine in the neostriatum, by blocking GABA in the ventral tegmental area, with bicuculline. They concluded that the catecholinergic system inhibits epileptic EEG activity and the expression of epileptic seizures, and produces an intense state of alertness, with fixed eyes, fear and isolationism (less epilepsy, more psychosis). Conversely, the induction of epileptic seizures through electrical stimulation led to a decrease in catecholamine levels and further psychotic manifestations. In turn, Sato et al. (1979) demonstrated that the progression of amygdala kindling to a generalized epileptic seizure is suppressed by the supersensitization of dopaminergic receptors by cocaine and apomorphine, and facilitated by the blocking of these receptors by neuroleptic drugs, such as haloperidol. Wada (1980) described that to reach the end point of a motor seizure becomes progressively more difficult the higher the rank on the phylogenetic scale, which is replaced by some other form of behavioral expression.

Together, these studies have demonstrated that electrical and pharmacological kindlings have completely different and antagonistic mechanisms, and have provided a theoretical basis for Landolt’s FN concept (Schmitz, 1998).

## New Concepts on Pathophysiological Mechanisms: Connectomics

Recent advances have brought a better understanding of the cellular processes involved in epileptogenesis, such as the functioning of ion channels and the role of neurotransmitters in the generation of epileptic seizures. At a systemic level, the development of new neuroimaging and electrophysiological techniques has reinforced the current concept that epilepsy is ultimately a disease of aberrant connections between neurons and neuronal groups. Above all, the abnormal neuronal synchronization characteristic of epilepsies can only be demonstrated at the level of neuronal networks (Engel et al., 2013). In this sense, structural connections (white matter tracts) were allowed to be demonstrated through Diffusion Tensor Imaging (DTI). On the other hand, in the study of functional connections, two tests started to be used: functional magnetic resonance imaging (fMRI) and invasive and non-invasive EEG. The association of both (EEG/fMRI) allowed access to more comprehensive information, with the spatial resolution of the fMRI being advantageous combined with the temporal resolution of the EEG. Thus, relevant neurophysiological events began to be studied functionally in spatial terms. The principle of functional connectivity (FC), enabled by the analysis with fMRI, is based on the concept that brain regions that show signal fluctuations at the same time are considered functionally related regions (Engel et al., 2013).

A recent work (Tong et al., 2019) studied the FC of interictal epileptiform discharges (IED) occurring in the hippocampus and amygdala of patients with unilateral temporal lobe epilepsy.

The authors analyzed, by EEG/fMRI, 261 events (133 on the left; 128 on the right) of 21 patients with unilateral TLE (10 on the left; 11 on the right). In short, they found that IEDs on the left exert greater influence in hippocampal-seeded networks. The IEDs on the right, in turn, exert greater influence on amydala-seeded networks. The main finding of the study was that left IED disconnects the ipsilateral hippocampus to the Default Mode Network (DMN). DMN deactivation at the time of the epileptiform event would explain the cognitive impairment associated with seizures in this region. The reward/emotion network also had its FC changed after the occurrence of IEDs. When occurring on the left, they preferentially altered the prefrontal limbic system (emotion); when on the right, they coactivated the reward system. The authors interpreted this last finding as a possible mechanism for FN.

### Resting-State Networks in TLE

The resting-state network is a specific group of brain structures that work synchronously during rest, and are modulated by specific functional tasks. They are involved in the control of higher cortical functions, such as: awareness, cognition, affective behavior and attention. TLE affects the resting-state network, and can be considered a disease of that system (Cataldi et al., 2013). In the normal brain, there are two large groups of neural networks (Biswal et al., 1995): Group 1 is involved in sensory and motor processes, and is formed by the visual network, auditory network, and sensoriomotor network; Group 2 is involved with higher cortical functions, and is formed by DMN, attention networks (dorsal and ventral), salience network, reward/emotion network, and language networks.

DMN is formed by the prefrontal cortex, medial, lateral and inferior parietal cortices, precuneus and cerebellum. It is activated by cognitive tasks, memory retrieval, envisioning of the future, theory of mind and in expressing moral judgments. On the other hand, DMN has its activity reduced in response to external challenges, goal-oriented tasks and in TLE. The decrease in DMN connectivity is proportional to the duration and severity of TLE and may reflect cumulative structural changes. On the other hand, DMN activation is decreased concomitantly with the appearance of interictal discharges on the EEG. This deactivation occurs mainly in the posterior cingulate, precuneus and bilateral frontal and parietal lobes (Laufs et al., 2007).

The reward/emotion network can be divided into two main systems: reward and emotional systems (Tomasi and Volkow, 2011). The reward system is centered on the mesolimbic dopaminergic system (ventral tegmentum and its projections). The emotional system is primarily based on the prefrontal limbic system. The common structure of these two systems is the amygdala, which serves as a functional hub for identifying the reward/emotion system by fMRI studies (Etkin et al., 2015).

In a recent meta-analysis, Doucet et al. (2013) concluded that hippocampus-seeded FC presents greater alterations in patients with left TLE, while amygdala-seeded FC is more altered in patients with right TLE. In this sense, epileptiform discharges on the left exert a recruitment of DMN by the left hippocampus, while epileptiform discharges on the right increase the FC of the right amygdala, exerting their actions above all on the reward/emotion network. Therefore, left epileptiform discharges can alter emotional regulation through disturbances of the prefrontal limbic system, and, conversely, the FC between the right amygdala and the reward system increases immediately after right epileptiform discharges (Di et al., 2017).

The transitory coactivation of the reward system after right IEDs could explain the phenomenon of FN, and the fact that epileptic activities and psychic symptoms are bidirectional conditions (Kanner, 2011). This hypothesis is supported by practical examples, like the use of electroconvulsive therapy for the treatment of depression (Tong et al., 2019).

### Patterns of Altered Functional Connectivity in the TLE

Pittau et al. (2012) studied FC between mesial temporal structures and resting-state networks. The authors selected EEG/fMRI data, without EEG discharges, from 23 patients with mTLE (16 on the right, and 7 on the left). Data were compared to 23 healthy control subjects, matched for age, gender and hemispheric dominance. The study had 4 main findings: (1) patients with mTLE (right or left) had a significant decrease in FC from healthy mesial temporal structures contralateral to the epileptic focus with regions of DMN and ventromedial limbic prefrontal structures; (2) decrease in FC contralateral to the focus with the diseased hippocampus, and DMN and ventromedial limbic prefrontal structures; (3) the amygdala of the healthy and diseased sides present the same alterations as the hippocampal FC; and (4) FC altered between mesial temporal structures and the reticular formation. The altered connectivity between mesial temporal structures and the mesolimbic network could explain the psychiatric disorders in patients with TLE, even in the absence of epileptiform discharges.

### EEG and Psychiatric Disorders in Temporal Lobe Epilepsy

One of the most controversial issues regarding the association between TLE and psychiatric comorbidities refers to electroencephalographic characteristics of this association. In a recent meta-analysis on the subject (Lourenço et al., 2020), the authors concluded that there are few published studies, with contradictory conclusions regarding the laterality and rate of epileptiform discharges, as well as the psychiatric pathologies found (psychosis × depression).

Bragatti et al. (2009) found an association between epileptiform discharges occurring in the left hemisphere with a three times greater chance of mood disorders, and, when infrequent (<1/min), these were statistically associated with mood disorders (Bragatti et al., 2014). Based on the fact that epileptiform discharges are associated with alterations in blood flow and brain metabolism (Rodin, 2009), the authors proposed that the low rate of discharges found in patients with mood disorders could be a biomarker for the diagnosis of depression.

Periodicity of epileptiform discharges was identified as a marker of clinical severity, characterized by refractoriness to drugs and severe psychiatric comorbidities (San-Juan et al., 2013). This statement was supported by another study, which concluded that patients with infrequent discharges (< 60/h) have a low percentage of refractoriness, with little or no presence of comorbidities (Napolitano et al., 2021).

Using invasive electrodes, Kuba et al. (2012) described two cases: in the first case, during the period of postictal psychosis (PP), the patient’s EEG showed rhythmic slow waves and “abortive” spike-slow wave complexes in the right hippocampus and neocortex. In the second patient, the EEG showed periodic triphasic waves in the left anterior cingulate gyrus, in the PP period (different from the ictal pattern found in these patients). The phenomenon of FN was also supported by the study of two patients with TLE who developed prolonged PP. During this period, repeated EEGs did not demonstrate epileptiform discharges, which eventually returned after remission of psychotic symptoms (Akanuma et al., 2005). However, Schulze-Bonhage and van Elst (2010) described two cases in which the rate and distribution of spikes did not change during the PP.

## Epilepsy as a Neurodegenerative Disorder: Gray Matter Structural Abnormalities in the Limbic System Associated With Psychiatric Disorders

Using three different neuroimaging tests (voxel-based morphometry, diffusion tensor imaging, and probabilistic tractography), Bonilha et al. (2010) found an important reduction in regional gray matter volume and fractional anisotropy in the limbic and perihippocampal areas of patients with mTLE. There was also a reduction in probabilistic tractography in the limbic areas of these patients. A significant relationship between loss of hippocampal connections and atrophy of regional gray matter volume was found involving the putamen, pallidum, middle and inferior temporal areas, amygdala and cerebellar hemisphere, suggesting that extrahippocampal atrophy in patients with mTLE may be related to hippocampal deafferentation.

A recent work studied the long-term influence of the progression of hippocampal atrophy, over time, on the dynamics of interictal epileptiform discharges in patients with TLE (Brito et al., 2021). The authors classified the EEGs into four groups: (1) ipsilateral discharges (to hippocampal sclerosis), (2) bilateral discharges, (3) contralateral discharges, and (4) normal EEG. Duration of epilepsy and EEG groups were statistically related. There was an increase in ipsilateral discharges and a decrease in normal EEGs over time. Contralateral discharges remained stable, which may reflect early abnormalities rather than progression of epilepsy. Thus, psychiatric comorbidities could be related to contralateral discharges to hippocampal sclerosis.

On the other hand, cortical thickness abnormalities may be related to other symptoms in the patient with TLE, in addition to epileptic seizures. The severity of depressive symptoms in TLE patients has been associated with a thickening of the orbitofrontal cortex, a limbic region of emotional processing strongly interconnected with mesial temporal structures (Butler et al., 2012).

## Discussion

The phenomenon of FN, established over 60 years ago, is composed of some dogmas and many controversies. Initial studies, with experimental animal models, in trying to reproduce the scenarios of TLE and its psychiatric comorbidities, demonstrated that there is a real antagonism between the phenomena of epileptic origin compared to psychiatric manifestations resulting from dysfunctions established in limbic circuits, whether by electrical stimulation, or by pharmacological manipulation of these circuits. In principle, this pathophysiological antagonism would reinforce the general thesis of the phenomenon of FN. However, there are two difficulties contrary to this theory: (1) there is no evidence that the kindling phenomenon occurs in humans; and (2) in animals, the phenomenon is not due to a structural lesion in the temporal lobe, although in humans, temporal lobe epilepsy is often caused by some injury disorder (Adamec, 1990).

The coactivations and deactivations of resting-state networks, amply demonstrated by EEG/fMRI studies, can explain the phenomenon of FN and also the bidirectionality between epilepsy and psychiatric comorbidities, even in the absence of epileptiform discharges. However, there is an alternative hypothesis to explain the FC phenomena found so far: the changes occurring in the DMN would facilitate the emergence of IEDs (Steriade, 2005). This hypothesis has already been proven in the case of generalized discharges and their activations with spindles and awakening. The thalamus would play a role in the propagation of generalized discharges, as well as the hippocampus in the propagation of IEDs in TLE. Both phenomena would result in antidromic deactivation in the DMN.

The biggest controversies concern the influence of IEDs on the psychiatric manifestations of patients with TLE. There are controversies regarding the lateralization and amount of discharges, which would be a biomarker for mood disorders, as well as regarding the disappearance of discharges at the time of onset of psychotic symptoms, which would support the phenomenon of FN. Correlations between the duration of epilepsy and possible associated progressive degenerative changes, installed in key regions for emotional control, and that should be better explored.

Only prospective studies, with homogeneous populations, using structured questionnaires, and applying the new methodology of EEG/fMRI coupling, will be able to shed more light on the FC map of different neuronal networks and its relationship with the epileptic phenomenon present in different regions of the cerebral cortex. Future studies may more clearly unveil the real relationships that exist between IED and resting-state networks, thus explaining the pathophysiology of FN.

## Author Contributions

The author confirms being the sole contributor of this work and has approved it for publication.

## Conflict of Interest

The author declares that the research was conducted in the absence of any commercial or financial relationships that could be construed as a potential conflict of interest.

## Publisher’s Note

All claims expressed in this article are solely those of the authors and do not necessarily represent those of their affiliated organizations, or those of the publisher, the editors and the reviewers. Any product that may be evaluated in this article, or claim that may be made by its manufacturer, is not guaranteed or endorsed by the publisher.
